# Four‐Dimensional Deoxyribonucleic Acid–Gold Nanoparticle Assemblies

**DOI:** 10.1002/anie.202007616

**Published:** 2020-07-28

**Authors:** Ming Luo, Mingjun Xuan, Shuaidong Huo, Jilin Fan, Gurudas Chakraborty, Yixi Wang, Hui Zhao, Andreas Herrmann, Lifei Zheng

**Affiliations:** ^1^ Institute of Fundamental and Frontier Sciences (IFFS) University of Electronic Science and Technology of China (UESTC) 610054 Chengdu China; ^2^ DWI-Leibniz Institute for Interactive Materials Forckenbeckstr. 50 52056 Aachen Germany; ^3^ Institute of Technical and Macromolecular Chemistry RWTH Aachen University Worringerweg 2 52074 Aachen Germany; ^4^ School of Chemical Engineering Sichuan University 610055 Chengdu China

**Keywords:** dissipative assembly, DNA, gold, nanoparticles, nanorods

## Abstract

Organization of gold nanoobjects by oligonucleotides has resulted in many three‐dimensional colloidal assemblies with diverse size, shape, and complexity; nonetheless, autonomous and temporal control during formation remains challenging. In contrast, living systems temporally and spatially self‐regulate formation of functional structures by internally orchestrating assembly and disassembly kinetics of dissipative biomacromolecular networks. We present a novel approach for fabricating four‐dimensional gold nanostructures by adding an additional dimension: time. The dissipative character of our system is achieved using exonuclease III digestion of deoxyribonucleic acid (DNA) fuel as an energy‐dissipating pathway. Temporal control over amorphous clusters composed of spherical gold nanoparticles (AuNPs) and well‐defined core–satellite structures from gold nanorods (AuNRs) and AuNPs is demonstrated. Furthermore, the high specificity of DNA hybridization allowed us to demonstrate selective activation of the evolution of multiple architectures of higher complexity in a single mixture containing small and larger spherical AuNPs and AuNRs.

## Graphical abstract

**Golden ratio**: Four‐dimensional gold (Au) nanostructures were fabricated by a dissipative assembly strategy. The dissipative character of the system was realized by utilizing deoxyribonucleic acid (DNA) as the fuel and exonuclease III (Exo III) as the energy‐dissipating unit, to generate AuNPs functionalized with short single‐stranded DNA oligonucleotides. The assemblies possess surface plasmonic resonance properties.

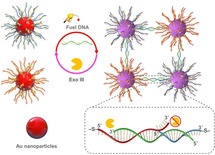

Since Mirkin and Alivisatos pioneered the utilization of oligonucleotides to organize gold nanoobjects by exploiting specific base paring, this concept has rapidly attracted a lot of attention in the field of nanoscience and flourished in a large number of deoxyribonucleic acid (DNA)‐based colloidal assemblies, including amorphous clusters,^1^ well‐defined finite molecule‐like,^2^ and infinite crystalline‐like architectures.^3^ DNA gold nanostructures are undoubtedly one of the most important classes of nanomaterials with many potential applications ranging from sensing to optoelectronics.^4^ Among these structures, the vast majority are non‐reconfigurable static assemblies, which often exhibit unique physiochemical properties. Alternatively, since hybridization of DNA is programmable and reversible, dynamic DNA–Au nanosystems were designed in which different states of the assemblies can be manipulated by strand‐displacement reactions.^5^ In these systems, all of the steady states are generally thermodynamically favorable and remain infinitely stable, unless external triggers are introduced into the system. Hence, self‐regulation as well as autonomous time programmability are not yet feasible with these switchable assemblies.^6^

Such dissipative assembly strategies rely on the continuous consumption of energy to sustain functional structures that are not at thermodynamic equilibrium, and thus promise a novel approach that could readily result in materials with life‐like properties.^7^ The underlying mechanism of this approach consists of at least two antagonistic reactions or processes: one energetic uphill reaction or process consuming fuel to form the transient structure, and another one dissipating the energy to render the system back to its original state. Importantly, the kinetic parameters of activation (v1) and deactivation (v2) must be properly tuned (that is, v1≫v2) to ensure the formation of the metastable intermediate. In the past few years, this strategy has led to a broad spectrum of materials, including transient self‐healing hydrogels^8^ and colloidal assemblies,^9^ temporary nanoreactors and catalysts,^10^ dissipative membrane channels,^11^ transient molecular cargo‐loading devices,^12^ among others.^13^ Although the dissipative clustering of gold nanoparticles (AuNPs) has been reported,^14^ more sophisticated and well‐defined out‐of‐equilibrium nanostructures with programmability of the time domain and richer physiochemical properties remain challenging.^15^

To achieve such four‐dimensional (4D) assemblies of the important class of DNA–Au nanostructures, we herein present the implementation of a dissipative assembly principle in respect to DNA‐fueled gold nanoobjects (Au‐nanoparticles (AuNPs) and gold nanorods (AuNRs)), leading to the dissipative clustering of AuNPs accompanied by the evolution of surface plasmonic resonance properties. This further results in the first examples of well‐defined out‐of‐equilibrium core–satellite architectures with programmable life times. Moreover, the high specificity of DNA hybridization further allows us to selectively activate the transient assembly in systems of higher complexity.

In previous studies, RNA strands were utilized as a fuel to drive the formation of polymeric colloid assemblies,9d DNA nanotubes,13g, 13h and a molecular device.^12^ The advantage of using RNA fuel within mixed RNA/DNA assemblies is the easy control over the selectivity of fuel consumption by RNases while maintaining DNA strands as structural elements within the assemblies. However, RNA is expensive and chemically less stable than DNA due to the presence of an extra hydroxyl group that renders this biomacromolecule more susceptible to hydrolysis under a wide variety of conditions. This fact not only limits the use of environments where such RNA fuel‐based assemblies can be applied, as in biological media, but might also cause undesired fuel consumption. Therefore, in our design, we decided to use DNA as fuel and further took advantage of the fast DNA hybridization kinetics and the tunable enzymatic DNA digestion rates to orchestrate the time domain of the DNA‐based gold‐nanoparticle assemblies. The transient structure formation is induced by the introduction of DNA fuel strands, which bridge the two corresponding complementary sequences attached on AuNPs and/or AuNRs. As the fuel‐consuming enzyme, we employed exonuclease III (Exo III), which specifically degrades double‐stranded DNA with 3′‐blunt ends or 3′‐overhangs that are shorter than four bases.^16^ Thanks to these properties, we could selectively digest the fuel strand and render the DNA strands attached to Au nanoobjects stable by introducing five base 3′‐overhangs. Another important feature of Exo III is that it starts to digest fuel DNA only when it is hybridized with the DNA strands on AuNPs. As a result, we were able to establish an internal feedback mechanism to connect the formation of transient structures and fuel consumption (Figure 1).

**Figure 1:**
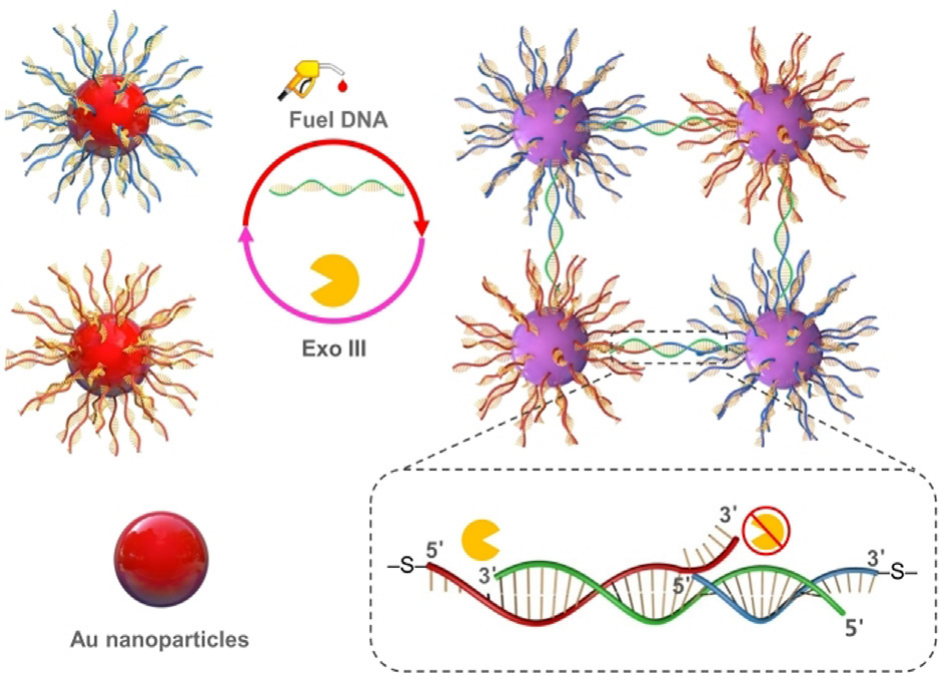
Representation of the dissipative clustering of spherical AuNPs using a DNA strand as the fuel and Exo III as the energy‐dissipating element. DNA hybridization and the associated fuel strand digestion act as antagonistic processes to induce the assembly and disassembly of AuNPs, respectively.

Initially, we verified the selectivity of fuel strand digestion by Exo III under non‐dissipative conditions. The fuel strand consisting of 24mer DNA was hybridized with two corresponding DNA sequences without being attached to AuNPs or AuNRs. The as‐prepared double strands were incubated with Exo III and were then investigated by denaturing polyacrylamide gel electrophoresis (PAGE). The gel image clearly revealed that Exo III only digested the fuel DNA without affecting the integrity of the other two sequences (Figure 2 a, denaturing PAGE). Moreover, when another portion of the fuel strand was added to the digested solution, the double‐stranded DNA configuration was regenerated (Figure 2 a, native PAGE). Notably, the remaining band (line 5, native PAGE), which cannot match DNA1 and DNA2, probably corresponds to the complex of DNA 2 and residues of digested fuel DNA 3. These results represent a sound proof that the sequence composition of the DNA strands fulfill the desired purpose to achieve transient gold nanostructure formation.

**Figure 2:**
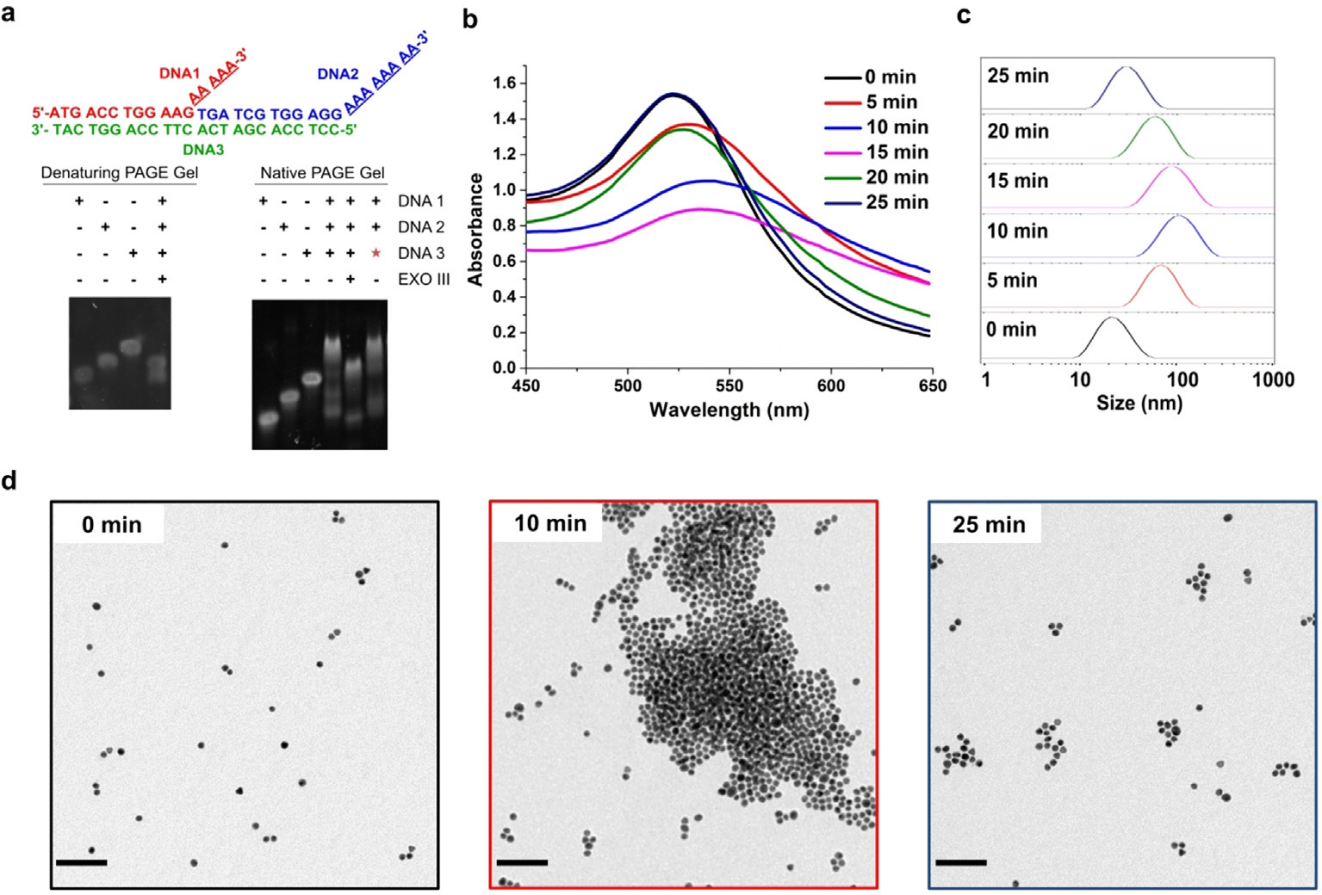
a) Gel electrophoretic analysis of fuel strand digestion by Exo III under non‐dissipative conditions. DNA 1 and 2 represent the sequences that will be attached to gold while DNA 3 represents the fuel strand (★: another portion of the fuel DNA 3 was added to the digested solution). b) UV/Vis absorption spectra measured every 5 min after the addition of fuel DNA (0.04 nmol) in the presence of 8 U μL^−1^ enzyme. c) DLS measurements under the described conditions. d) TEM images acquired before and after the addition of fuel DNA (0.04 nmol) at 10 and 25 min in the presence of 8 U μL^−1^ enzyme; scale bar=100 nm.

Subsequently, we aimed to create an autonomously aggregating AuNP system, which is characterized by evolving amorphous aggregates without external intervention. The dissipative clustering of DNA‐modified AuNPs with a diameter of 15 nm (15‐AuNPs) promoted by DNA fuel strands and the presence of Exo III was initially investigated by UV/Vis absorption spectroscopy. At the beginning of the experiment, the absorption spectrum showed a gradual redshift from 522 to 540 nm with decreasing absorbance intensity indicating that the 15‐AuNPs aggregated by the addition of the complementary fuel strand. After 15 minutes, the absorption spectrum shifted toward the blue end of the spectrum and the absorption maximum increased, indicating that the clusters started to dissemble due to the consumption of the fuel strands. After an overall reaction time of 25 minutes, the initial spectrum (0 min) with an absorption maximum at 522 nm was recovered (Figure 2 b). The evolution of UV/Vis spectra clearly indicates that transient AuNP aggregates were formed and finally disassembled due to the digestion of fuel DNA by the enzyme Exo III, and it demonstrates the capability of our approach for autonomously tuning the optical properties of plasmonic nanostructures with temporal programmability. This size evolution of AuNP assembly was further corroborated by following the process with dynamic light scattering (DLS) (Figure 2 c) and transmission electron microscopy (TEM, Figure 2 d). DLS traces showed that the size of the clusters increased during the first 15 minutes and after that decreased to the original state. Similarly, TEM images at different time points showed the formation of transient extended colloidal aggregates at 10 minutes that disaggregate into very small clusters at 25 minutes. Notably, some small aggregates were always observed by TEM at the end of each cycle, which might result from residual fuel DNA in the system.

We further used recovery time as a parameter to quantify the dependence of the dissipative clustering process on the enzyme concentrations and the fuel concentrations. Herein, we defined recovery time as the time interval during which the dissipative assembly system was recovered to its initial state, as indicated from the absorption spectrum (the starting point is the time when the DNA fuel was added into the system). Upon increasing the concentration of Exo III from 2 to 8 U μL^−1^, with a fixed amount of the fuel strand (0.04 nmol), the recovery time decreased from 50 to 25 minutes (Figure 3 a; for additional UV/Vis spectral data see the Supporting Information, Section 2). In contrast, when the amount of the fuel DNA was increased from 0.04 to 0.16 nmol with a fixed Exo III concentration of 6 U μL^−1^, the recovery time increased from 35 to 180 minutes. Figure 3 b showed a linear relationship between recovery time and the concentration of fuel DNA that were applied. Finally, the possibility of inducing transient assembly of the 15‐AuNPs in consecutive cycles was also tested through the repetitive addition of a constant amount of DNA fuel (0.04 nmol) into the system. It was observed that, in the presence of 8 U μL^−1^ enzyme at least five assembly/disassembly cycles were obtained. However, after each cycle the recovery time gradually increased and the absorbance at 522 nm decreased (Figures 3 c,d; for additional analysis of UV/Vis spectral data see the Supporting Information, Section 2). This effect was more pronounced when lower concentrations of enzyme were applied (Figure 3 a), which might be explained by the limited stability of Exo III over time or the inhibition of the enzyme caused by the accumulation of nucleotide waste products.^12^

**Figure 3:**
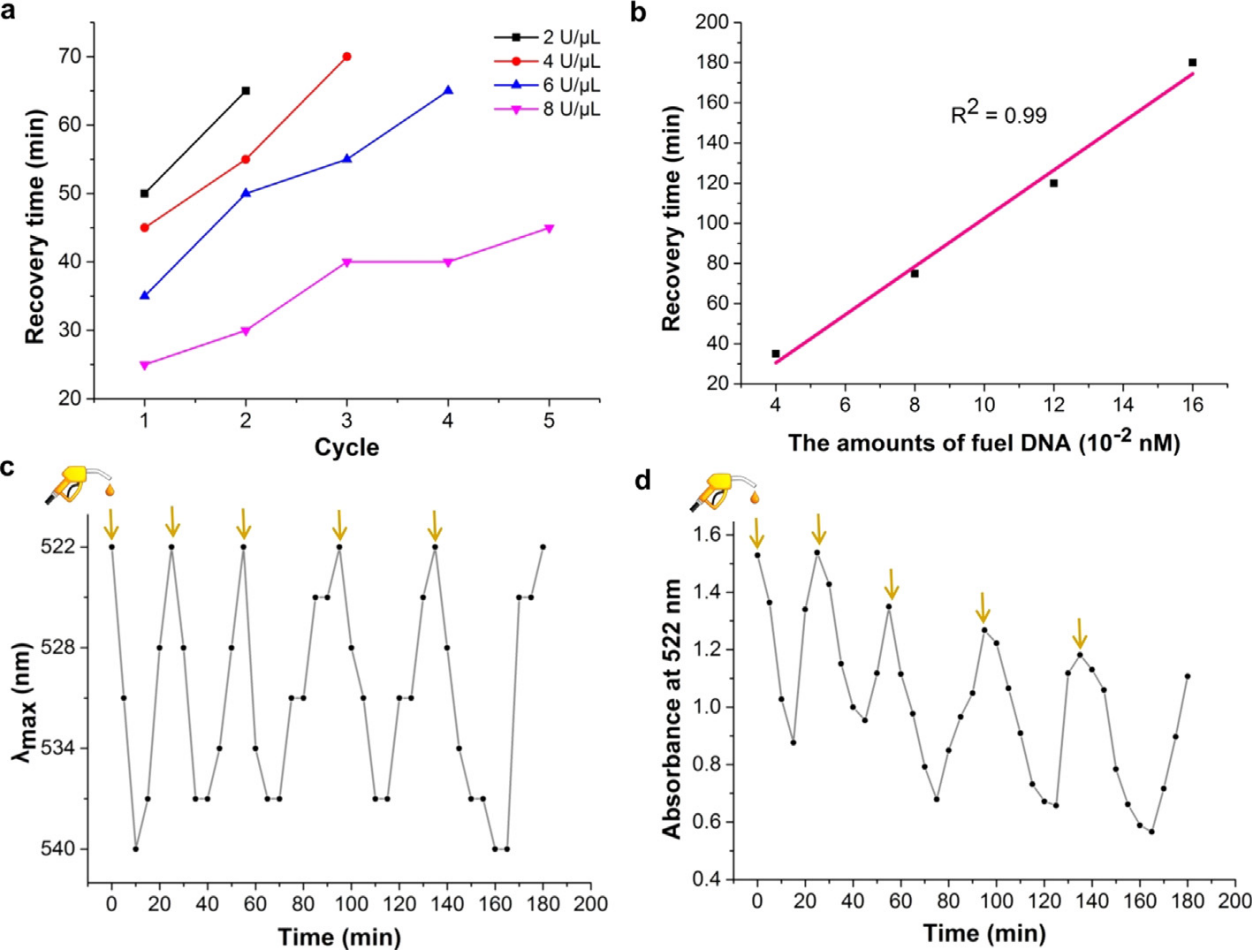
a) Recovery time for different cycles in the presence of 2, 4, 6, and 8 U μL^−1^ enzyme. b) Linear relationship between recovery time and the amount of fuel DNA (0.04, 0.08, 0.12, and 0.16 nmol) in the presence of 6 U μL^−1^ enzyme. c) Change of wavelength at the absorption maximum after sequential addition of the fuel DNA in the presence of 8 U μL^−1^ enzyme. d) Absorbance at 522 nm after sequential addition of the fuel DNA in the presence of 8 U μL^−1^ enzyme.

Encouraged by achieving 4D amorphous clusters, we attempted to exploit this molecular system to construct well‐defined dissipative AuNP architectures. For this purpose, we synthesized AuNRs modified with DNA sequences that are complementary to one half of the fuel strand. These AuNRs were dedicated to act as the core when involved in the formation of finite molecule‐like core–satellite structures with spherical 15‐AuNPs at the rim (Figure 4 a).^17^ These AuNRs were mixed with the corresponding spherical 15‐AuNPs encoded with the second half of the complement of the fuel strand in a ratio of 1:15. In the absence of Exo III, the addition of 1000 equivalents of linker DNA (in relation to AuNRs) into the mixture resulted in the formation of perfect core–satellite structures where the surfaces of AuNRs were fully decorated with 15‐AuNPs within 30 minutes (Figure 4 c, without enzyme). When the enzyme was present in the system, the TEM images taken at 15 minutes revealed that a lesser amount of the 15‐AuNPs were assembled on the AuNRs. In addition, we observed that at high Exo III concentration of 4 U μL^−1^, energy dissipation occurred at such a high rate that the steady core–satellite architectures could not be achieved (Supporting Information, Figure S26). Subsequently, we used TEM to investigate the structural evolution of the assemblies in the presence of 2U μL^−1^ enzyme. As shown in Figure 4 c, we found that in the first 30 minutes the 15‐AuNPs gradually assembled on AuNRs and afterwards started to dissociate from the AuNRs until reaching the initial state (for additional TEM images see the Supporting Information, Section 3). According to the TEM study, simple statistics were conducted by counting the number of 15‐AuNPs on AuNRs at different time points (Figure 4 b), which showed a clear dissipative assembly characteristic; that is, the satellite structures were evolving over time and maintained under the consumption of fuel until they disassembled again into the individual building blocks.

**Figure 4:**
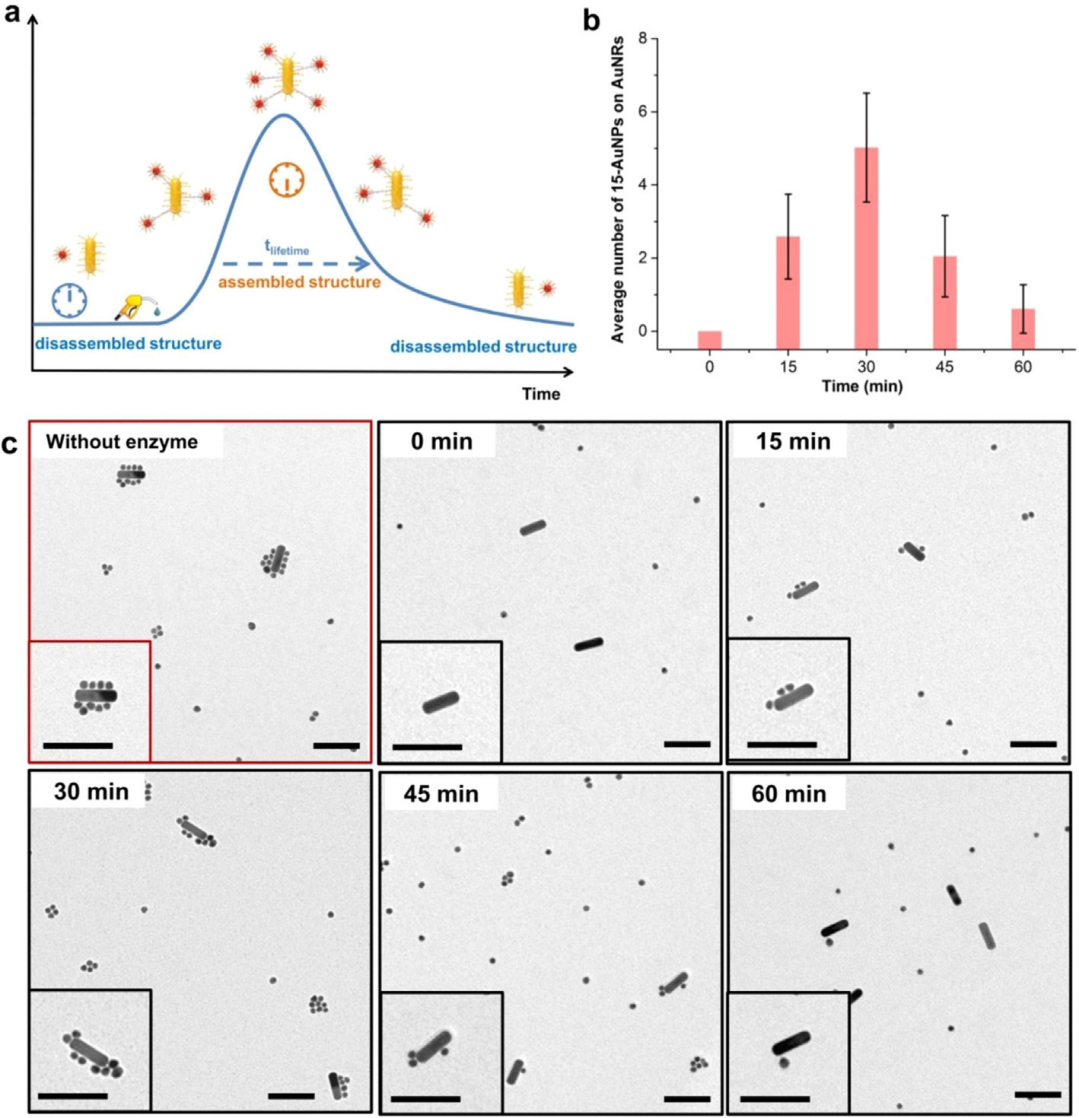
a) Representation of the structural evolution of the dissipative assembly of core–satellite architectures between 15‐AuNPs and AuNRs. b) Time‐dependent average number of 15‐AuNPs assembled on AuNRs in the presence of 2 U μL^−1^ Exo‐III. Average numbers for each time point were calculated for 68–82 aggregates from 16–21 TEM images. c) TEM studies on the structural evolution of core–satellite architectures; scale bars=100 nm.

An outstanding feature of the dissipative DNA assembly systems presented here is the sequence‐specific responsivity enabled by the high specificity of base pairing. This opens the opportunity to design transient DNA‐fueled architectures of higher complexity due to the fact that DNA sequences act as encoded or programmable fuel. To demonstrate this potential, we intended to build dissipative systems in which the building blocks can assemble into different structures within a certain time course upon activation with different DNA sequences (Figure 5 a). For this purpose, we synthesized a new type of DNA‐modified spherical AuNPs with 55 nm diameter (55‐AuNPs) that can act as the core to form round‐shaped particle‐on‐particle core–satellite architectures with the previously introduced smaller sized spherical 15‐AuNPs. The formation of this structure was induced by a new sequence of fuel DNA, which can hybridize with the two corresponding DNA sequences being attached to the two types of spherical AuNPs. Finally, three types of nanoobjects were combined together in the presence of Exo III as the common energy dissipation unit. Upon the addition of the first fuel DNA, we observed evolving dissipative core–satellite structures between 15‐AuNPs and AuNRs, as shown before. When the first fuel strand was consumed after 60 minutes, the same amount of the second fuel DNA was added to the solution, resulting in a similar structural evolution of core–satellites between 15‐AuNPs and 55‐AuNPs, as observed from TEM images (Figure 5 c; for additional TEM images, see the Supporting Information). We performed statistical analysis of the system, which showed two dissipative assembly cycles with no mutual interference (Figure 5 b). These experiments demonstrated the utility of DNA as a programmable fuel that can selectively activate dissipative assemblies of a certain structure in a mixture of different nanoobjects.

**Figure 5:**
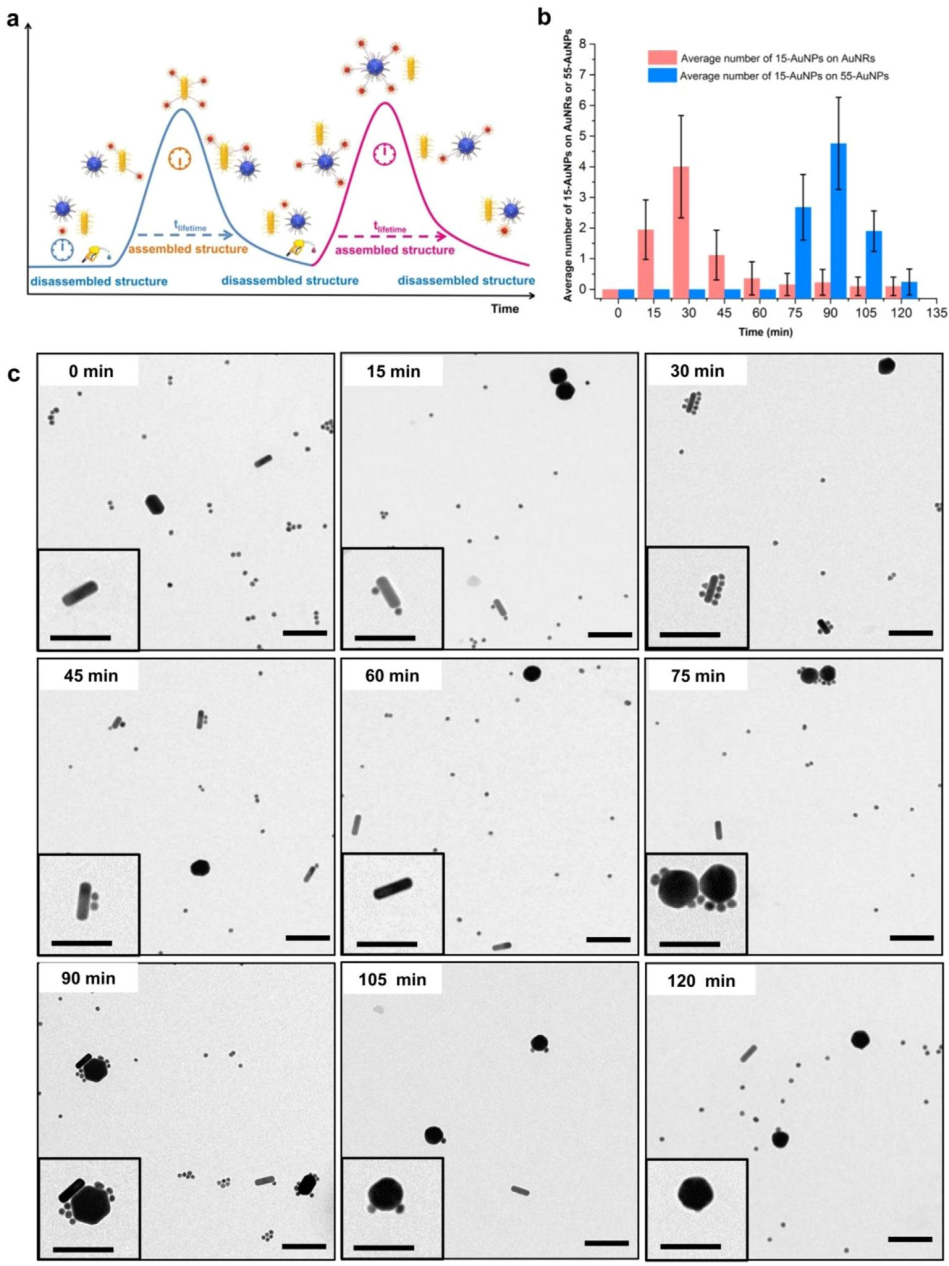
a) Representation of the consecutive structural evolution of dissipatively assembled core–satellite architectures involving 15‐AuNPs, 55‐AuNPs, and AuNRs. b) Time‐dependent average number of 15‐AuNPs assembled on AuNRs and spherical 55‐AuNPs in the presence of 3 U μL^−1^ Exo‐III. Average numbers for each time point were calculated for 35–43 aggregates from 12–16 TEM images. c) TEM studies showing the consecutive structural evolution of dissipative core–satellites activated by the sequential addition of two fuel DNA sequences; scale bars=100 nm.

In summary, we have presented a novel dissipative assembly system employing one of the workhorses of the field of nanoscience; that is, AuNPs functionalized with short single‐stranded DNA oligonucleotides. The dissipative character of the system was realized by utilizing DNA as the fuel and Exo III as the energy‐dissipating unit. Compared to other synthetic out‐of‐equilibrium structures, an important strength of the system presented here is the convenient application of the high specificity of DNA hybridization, which allowed us to demonstrate the selective activation of the evolution of multiple architectures of higher complexity in a single mixture. In this context, it is important to note that recent work has shown that the dynamic structural change of assembled AuNPs can regulate their interactions with living cells.^18^ This opens the path toward future DNA‐fueled nanosystems where structural evolution can be used to create functions such as sensing, self‐regulation of cellular interactions, or logic operations. Moreover, considering the longstanding interest in the dynamic control of surface plasmon resonance of AuNPs, we believe that this work will inspire future design of DNA‐based out‐of‐equilibrium plasmonic nanosystems with autonomous behavior and spatiotemporal controllability.^19^

## Conflict of interest

The authors declare no conflict of interest.

## Supporting information

As a service to our authors and readers, this journal provides supporting information supplied by the authors. Such materials are peer reviewed and may be re‐organized for online delivery, but are not copy‐edited or typeset. Technical support issues arising from supporting information (other than missing files) should be addressed to the authors.

Supplementary
