# Supplementary material for: Four‐Dimensional Deoxyribonucleic Acid–Gold Nanoparticle Assemblies
Source: Angew Chem Int Ed Engl. 2020 Jul 28;59(39):17250–5. doi: 10.1002/anie.202007616 (PMC7540408; doi:10.1002/anie.202007616)
Supplement: Supplementary file 1 — Supplementary [file ANIE-59-17250-s001.pdf]

Angewandte Chemie
Eine Zeitschrift der Gesellschaft Deutscher Chemiker

Supporting Information

Four-Dimensional Deoxyribonucleic Acid–Gold Nanoparticle Assemblies

Ming Luo, Mingjun Xuan, Shuaidong Huo, Jilin Fan, Gurudas Chakraborty, Yixi Wang, Hui Zhao, Andreas Herrmann,* and Lifei Zheng**

anie_202007616_sm_miscellaneous_information.pdf

Contents

1. Materials and experimental procedures S3

2. UV-Vis spectra data and analysis for AuNPs dissipative assemblies S7

3. TEM studies on the structural evolution of core-satellite architectures S19

4. References S26

1. Materials and experimental procedures

Gold (III) chloride ($HAuCl_4$), sodium citrate tribasic, tannic acid, potassium carbonate, bis(p-sulfonatophenyl)phenylphosphine dihydrate dipotassium salt (BSPP), magnesium chloride ($MgCl_2$), sodium chloride (NaCl), Tween 20, and phosphate buffered saline (PBS) were purchased from Sigma-Aldrich (Germany). Exonuclease III and glycerin were purchased from Thermo Fisher Scientific (Germany). All DNA sequences used in this study were synthesized, labeled, and purified by Biomers (Germany).

Oligonucleotide sequences used in this study are as follows:

DNA 1	5'-ATG ACC TGG AAG AA AAA-3'
DNA 2	5'-TGA TCG TGG AGG AAA AAA AA-3'
DNA 3	5'-CCT CCA CGA TCA CTT CCA GGT CAT-3'
DNA 4	5'-SH−AA AAA A ATG ACC TGG AAG AA AAA-3'
DNA 5	5'- TGA TCG TGG AGG AAA AAA A−SH-3'
DNA 6	5'- TCA TAG TCG ACG AAA AAA A-SH-3'
DNA 7	5'-CGT CGA CTA TGA CTT CCA GGT CAT-3'

Methods

The morphology of the prepared AuNPs and AuNRs and self-assembled structures were examined using a Libra 120 Transmission Electron Microscope (Carl Zeiss, Germany) with 120 kV accelerating voltage. Optical absorption spectra were measured with a spectrophotometer (SpectraMax M3, Molecular Device). Hydrodynamic diameters of the AuNP clusters were determined by a Nano ZS Zetasizer (25 ℃, Malvern, England).

PAGE Gel Electrophoresis.

20% polyacrylamide gels were prepared. In case of preparing denaturing PAGE gels, 6 M urea was added. Electrophoresis was carried out at a voltage of 120 V for 150 min in TBE (1×) running buffer. Different samples were prepared as following:

Hybridized DNA (1+2+3): DNA1, DNA2, and DNA3 (3 μM) were mixed in a stoichiometric ratio in 50 mM Tris-HCl (pH 8.0) containing 1 mM $MgCl_2$. The mixture was annealed in a thermocycler by heating to 95 ℃ for 5 min and then cooling down to 20 ℃ with a stepwise temperature decrease rate of 5 ℃/min.

DNA (1+2+3) + Exo III: Exo III (2 U/μL) was added to the hybridized DNA(1+2+3) (3 uM) in 50 mM Tris-HCl (pH 8.0) containing 1 mM $MgCl_2$. The digestion mixture was incubated for 1 h at 25 ℃ and 1 μL of this mixture was directly subjected to PAGE analysis. The rest of the mixture was hybridized by another stoichiometric amount of DNA 3 in a thermocycler using the same program as mentioned above.

Synthesis and characterization of AuNPs and AuNRs

Synthesis of 15 nm Au@citrate nanoparticles**:** 15 nm Au@citrate NPs were prepared by the standard citrate reduction method as reported [1]. Briefly, 0.5 mL of $HAuCl_4 \cdot 3H_2O$ solution (1%, w/v) was mixed with 50 mL of Milli-Q water. The mixture was heated to boiling and then 1.5 mL of sodium citrate solution (1%, w/v) was added to the boiling solution quickly under vigorous stirring. The mixture was kept boiling for another 15-30 min and then was allowed to cool down to room temperature with stirring. Finally, the 15 nm Au@citrate NPs were obtained and used as seed solution to synthesize larger AuNPs with the diameter of 55 nm.

Synthesis of 55 nm Au@citrate nanoparticles: 55 nm Au@citrate NPs were synthesized using the seed growth method. Generally, 2.25 mL of 15 nm Au@citrate nanoparticle seed solution was mixed with 2.44 mL of $HAuCl_4 \cdot 3H_2O$ solution (10 mM) and then the mixture was diluted to 150 mL with Milli-Q water. Then, 100 mL of ascorbic acid solution (0.4 mM) was added to the above stirring solution at a rate of 10 mL/min employing a peristaltic pump. Finally, the 55 nm Au@citrate NPs in purple red were obtained. Nanoparticle concentration was determined by UV-vis absorbance *via* the Beer-Lambert law according to literature [3].

***Functionalization of AuNPs with DNA*:** Gold nanoparticles were functionalized with thiolated single-stranded DNA using a previously published method [4]. Briefly, gold nanoparticles were mixed with thiolated DNA strands in 750 µl of 0.01% Tween 20 according to the following table:

Nanoparticle size	15 nm		55 nm
Particle conc. (nM)	7.5 nM		1 nM
DNA sequence	DNA 4	DNA 5	DNA 6
DNA conc. (nM)	1000	1000	1000
Nanoparticle : DNA	1:133		1:1000

After 5 minutes incubation, 250 µl of sodium citrate buffer (100 mM of sodium citrate tribasic, pH 3.0) containing 0.01% Tween 20 was added. The mixture was incubated for 30 minutes at room temperature and subsequently purified by centrifugation for 6 min (55 nm AuNPs) and 30 minutes (15 nm AuNPs) at 4˚C. The centrifugation speed was set at 15,000 rpm. After removing the supernatant, DNA-functionalized nanoparticles were further washed using PBS buffer containing Tween 20 (0.01 %w/v) for two times and stored in the same buffer. The supernatant containing excess oligonucleotides were collected and the amount of DNA was determined according to the absorption spectra. From this, the amount of DNA loading on gold nanoparticles was estimated as shown Figure S1.

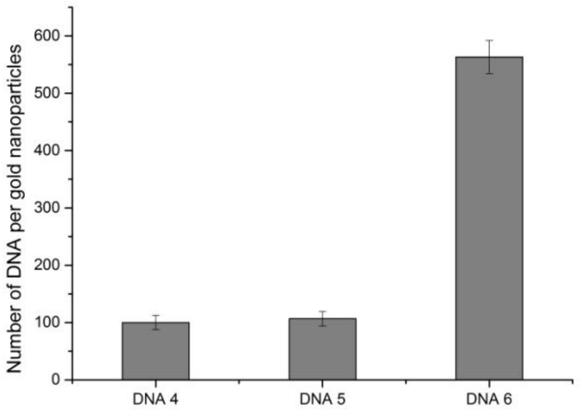

Figure S1. Quantification of DNA loading on gold nanoparticles.

***Synthesis of AuNRs*:** AuNRs were synthesized according to a published procedure[5]. First, CTAB-capped Au seeds were prepared by chemical reduction of $HAuCl_4$ with $NaBH_4$. 7.5 mL (0.1 M) CTAB was mixed with 250 μL (10 mM) $HAuCl_4$ and the volume was adjusted to 9.4 mL by adding MQ water. A 0.6 mL ice-cooled $NaBH_4$ aqueous solution (0.01 M) was added to the above mixture to initiate seed formation. The growth solution for AuNRs consisted of a mixture of 100 mL 0.1 M CTAB, 5 mL 0.01 M $HAuCl_4$, 0.8 mL 10 mM $AgNO_3$, 2 mL 0.5 M H_2SO_4 and 800 μL 0.1 M ascorbic acid. Growth of AuNRs was initiated by adding 240 μL seeds into the growth buffer and the temperature was kept constant at 30℃ during the full procedure. The AuNRs prepared here had a longitudinal surface plasmon resonance band centered at 754 nm in the presence of 100 mM CTAB.

***Functionalization of AuNRs with DNA*:** Firstly, 1×TBE and 0.1% SDS were mixed and shaken gently. HCl was used to adjust the mixture's pH to be 3.0. Then DNA5 was added to this mixture. Lastly, newly synthesized AuNRs were added to the solution. The ratio of DNA5 to AuNRs is 1000:1. After several minutes, the mixture was centrifuged (8 mins, 8000 rpm) for three times, the supernatant was discarded and the pellet was suspended in MQ water.

Standard procedure for dissipative clustering of 15-AuNPs in the presence of different amounts of enzyme

6 nM DNA4 functionalized 15-AuNPs and DNA5 functionalized 15-AuNPs were mixed in 100 μL 50 mM Tris-HCl buffer containing 200 mM NaCl, 4 mM $MgCl_2$ and 2 mg/mL BSA. Then Exo III was added with different concentrations (2 U/μL, 4 U/μL, 6 U/μL, and 8 U/μL). Finally, a fixed amount of fuel DNA 3 (0.04 nmol) was added into the system to initiate the dissipative clustering of AuNPs. UV-Vis absorption spectra were measured every five minutes after the addition of fuel DNA 3. When the absorption spectrum returned to its original state, another portion of fuel DNA 3 was added into the system and the next cycle of dissipative assembly started.

2. UV-Vis spectra data and analysis for AuNPs dissipative assemblies

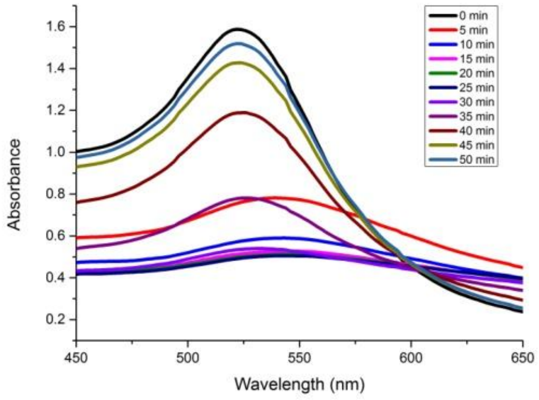

Figure S2. UV-Vis absorption spectra measured every five minutes after the addition of fuel DNA (0.04 nmol) in the presence of 2 U/μL enzyme (first cycle).

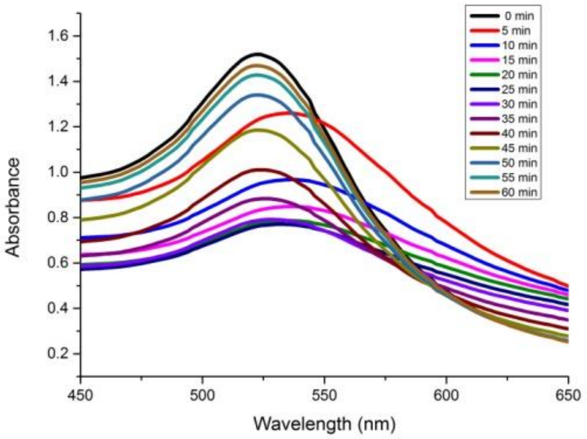

Figure S3. UV-Vis absorption spectra measured every five minutes after the addition of fuel DNA (0.04 nmol) in the presence of 2 U/μL enzyme (second cycle).

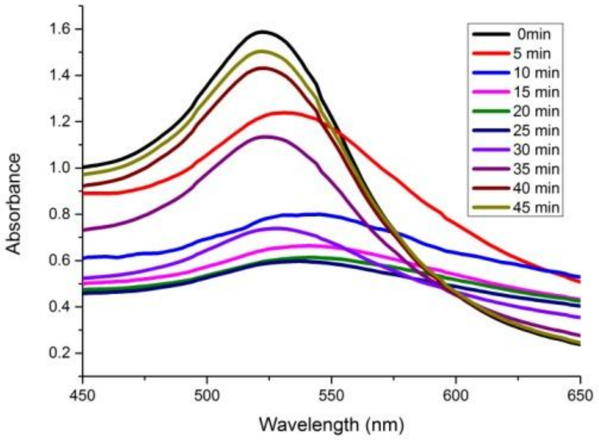

Figure S4. UV-Vis absorption spectra measured every five minutes after the addition of fuel DNA (0.04 nmol) in the presence of 4 U/μL enzyme (first cycle).

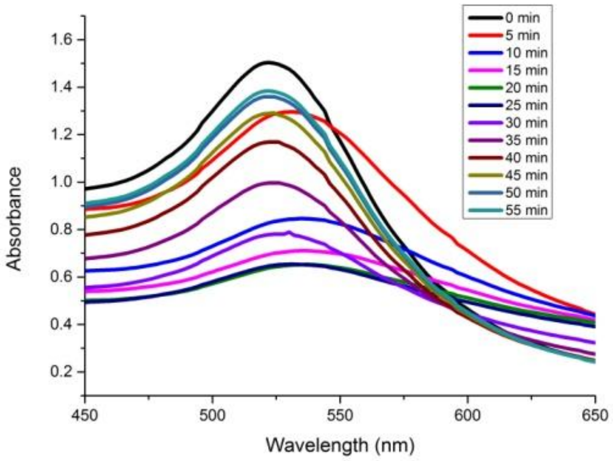

Figure S5. UV-Vis absorption spectra measured every five minutes after the addition of fuel DNA (0.04 nmol) in the presence of 4 U/μL enzyme (second cycle).

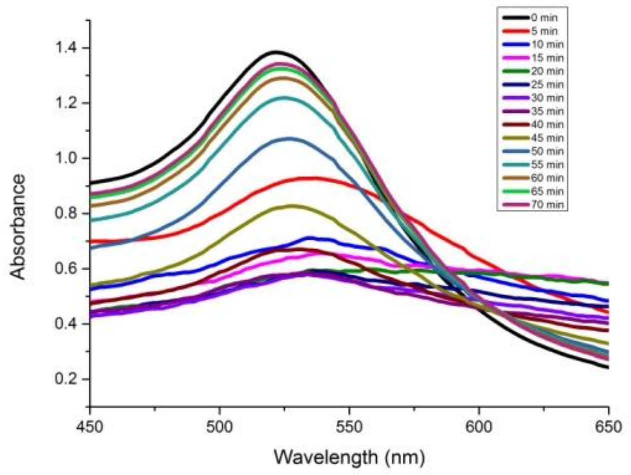

Figure S6. UV-Vis absorption spectra measured every five minutes after the addition of fuel DNA (0.04 nmol) in the presence of 4 U/μL enzyme (third cycle).

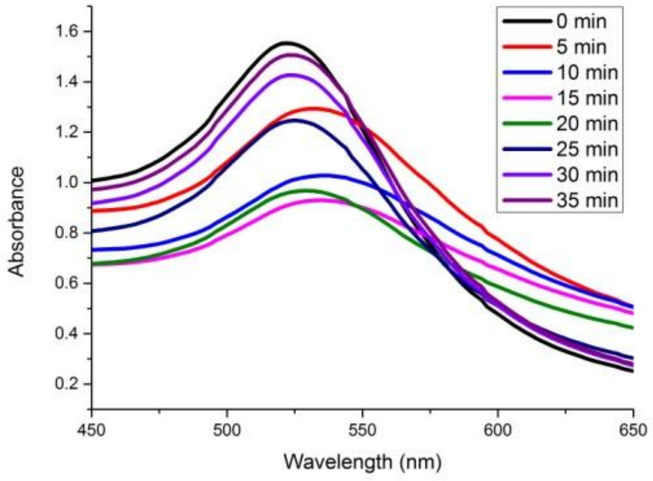

Figure S7. UV-Vis absorption spectra measured every five minutes after the addition of fuel DNA (0.04 nmol) in the presence of 6 U/μL enzyme (first cycle).

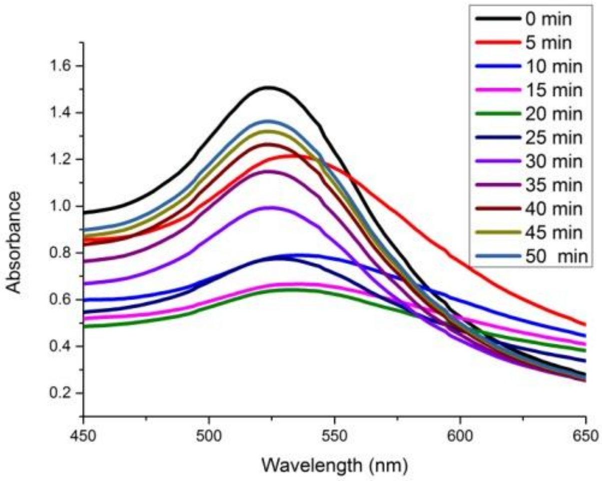

Figure S8. UV-Vis absorption spectra measured every five minutes after the addition of fuel DNA (0.04 nmol) in the presence of 6 U/μL enzyme (second cycle).

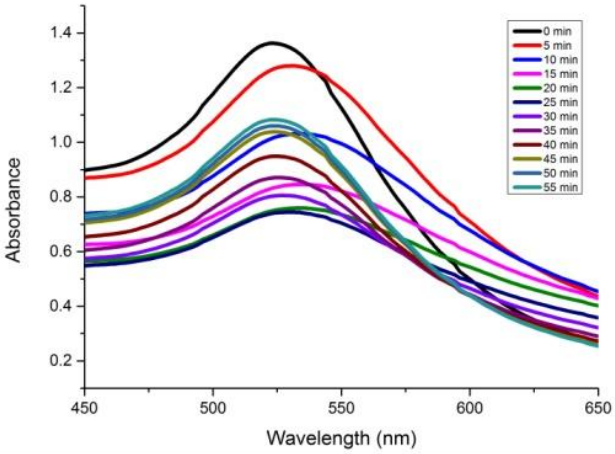

Figure S9. UV-Vis absorption spectra measured every five minutes after the addition of fuel DNA (0.04 nmol) in the presence of 6 U/μL enzyme (third cycle).

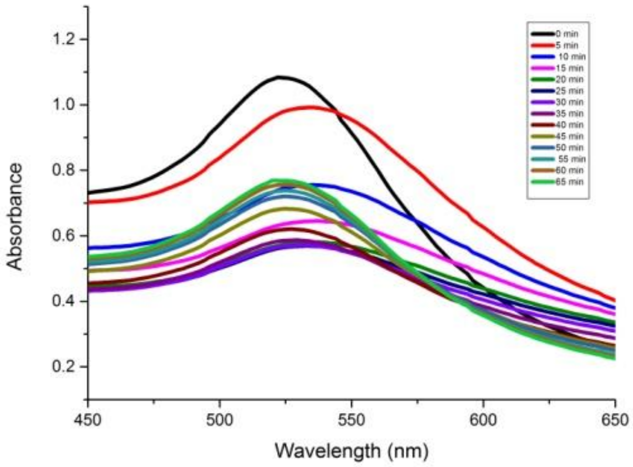

Figure S10. UV-Vis absorption spectra measured every five minutes after the addition of fuel DNA (0.04 nmol) in the presence of 6 U/μL enzyme (fourth cycle).

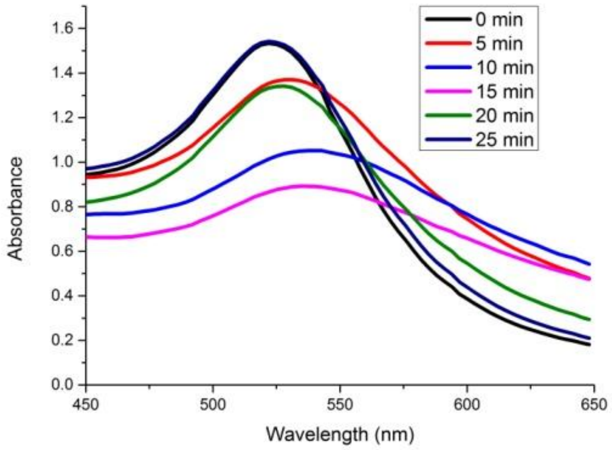

Figure S11. UV-Vis absorption spectra measured every five minutes after the addition of fuel DNA (0.04 nmol) in the presence of 8 U/μL enzyme (first cycle).

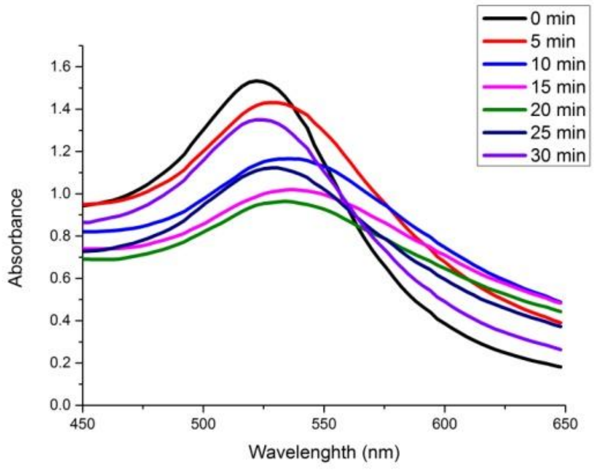

Figure S12. UV-Vis absorption spectra measured every five minutes after the addition of fuel DNA (0.04 nmol) in the presence of 8 U/μL enzyme (second cycle).

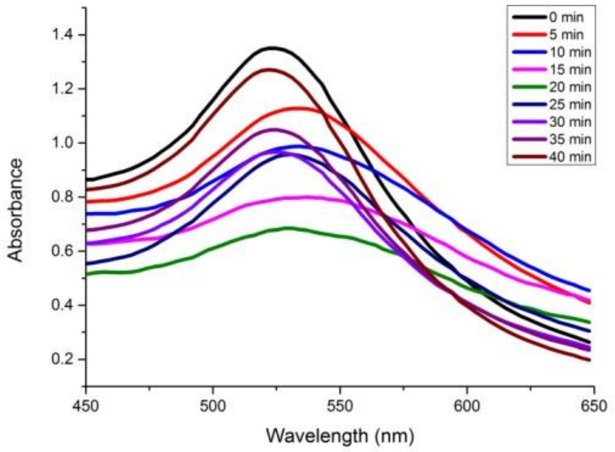

Figure S13. UV-Vis absorption spectra measured every five minutes after the addition of fuel DNA (0.04 nmol) in the presence of 8 U/μL enzyme (third cycle).

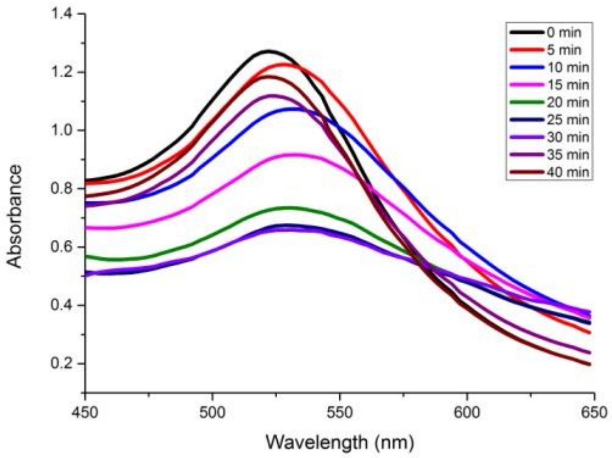

Figure S14. UV-Vis absorption spectra measured every five minutes after the addition of fuel DNA (0.04 nmol) in the presence of 8 U/μL enzyme (fourth cycle).

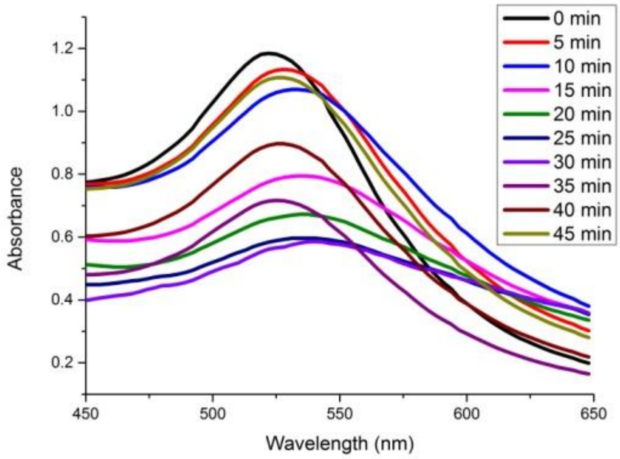

Figure S15. UV-Vis absorption spectra measured every five minutes after the addition of fuel DNA (0.04 nmol) in the presence of 8 U/μL enzyme (fifth cycle).

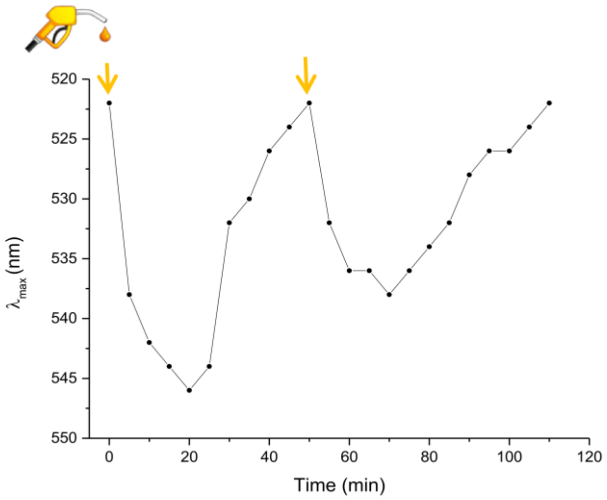

Figure S16. Change of wavelength at the absorption maximum after sequential addition of the fuel DNA in the presence of 2 U/μL enzyme.

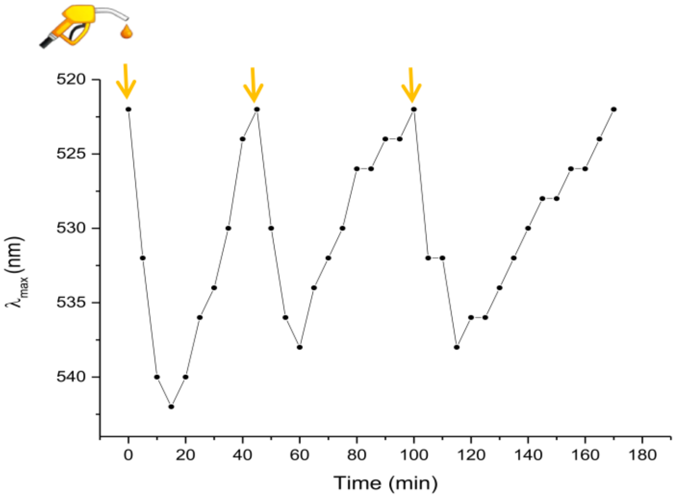

Figure S17. Change of wavelength at the absorption maximum after sequential addition of the fuel DNA in the presence of 4 U/μL enzyme.

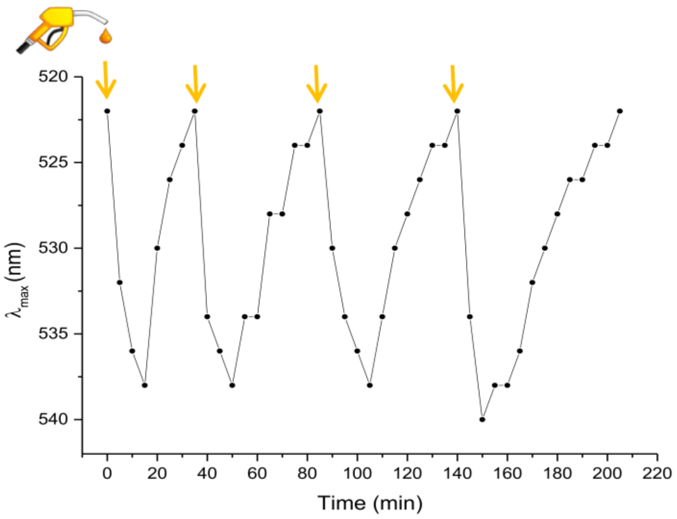

Figure S18. Change of wavelength at the absorption maximum after sequential addition of the fuel DNA in the presence of 6 U/μL enzyme.

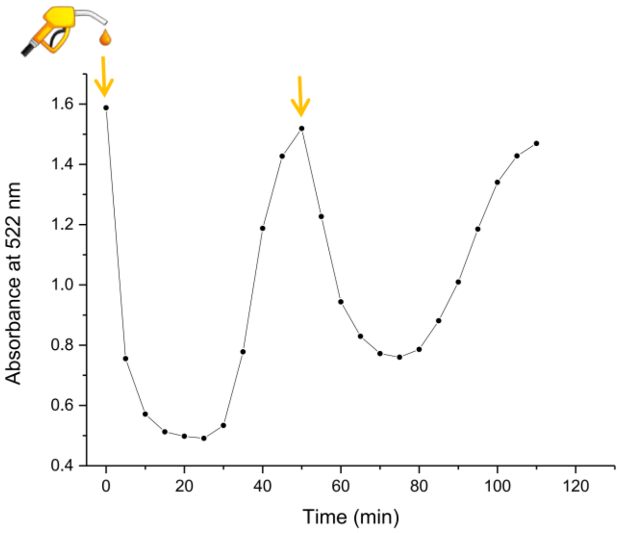

Figure S19. Absorbance at 522 nm after sequential addition of the fuel DNA in the presence of 2 U/μL enzyme.

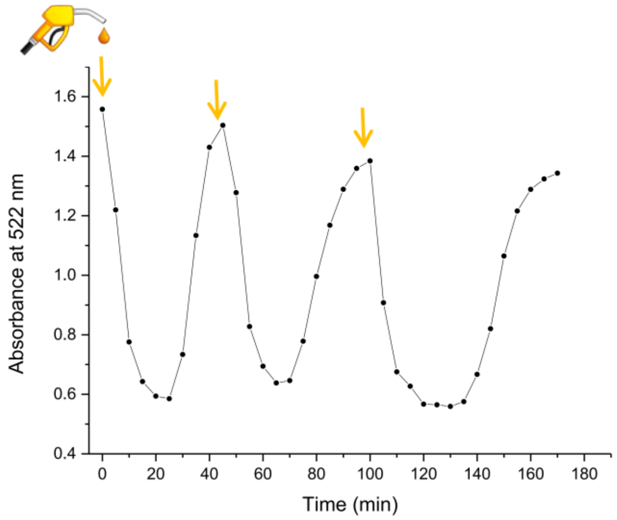

Figure S20. Absorbance at 522 nm after sequential addition of the fuel DNA in the presence of 4 U/μL enzyme.

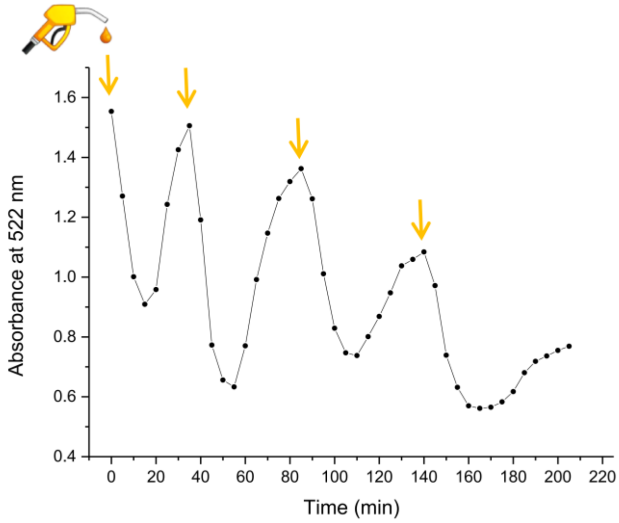

Figure S21. Absorbance at 522 nm after sequential addition of the fuel DNA in the presence of 6 U/μL enzyme.

Standard procedure for dissipative clustering of AuNPs in the presence of different amounts of fuel DNA

6 nM DNA functionalized DNA4-AuNPs and DNA5-AuNPs were mixed in 100 μL 50 mM Tris-HCl buffer containing 200 mM NaCl, 4 mM $MgCl_2$ and 2 mg/mL BSA. Then Exo III was added with a fixed concentration of 6 U/μL. Finally, different amounts of fuel DNA 3 (0.08 nmol, 0.12 nmol, and 0.16 nmol) was added into the

system to initiate the dissipative clustering of AuNPs. Again, UV-Vis absorption spectra were measured every five minutes to monitor the assembly processes.

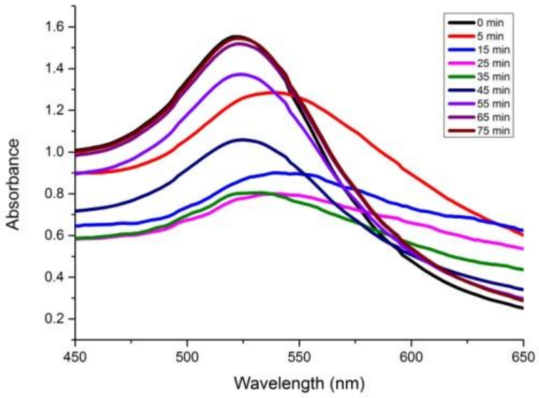

Figure S22. UV-Vis absorption spectra measured every five minutes after the addition of fuel DNA (0.8 μM) in the presence of 6 U/μL enzyme.

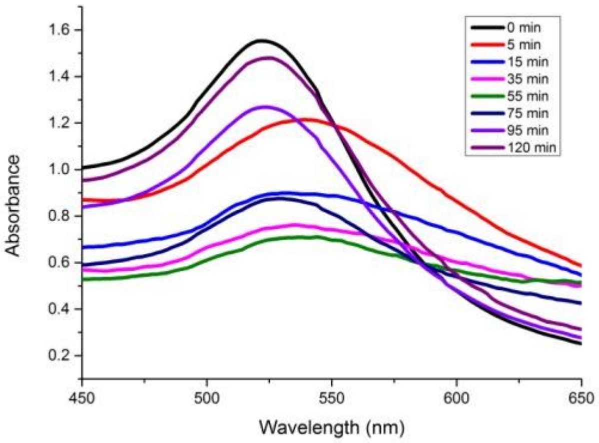

Figure S23. UV-Vis absorption spectra measured every five minutes after the addition of fuel DNA (1.2 μM) in the presence of 6 U/μL enzyme.

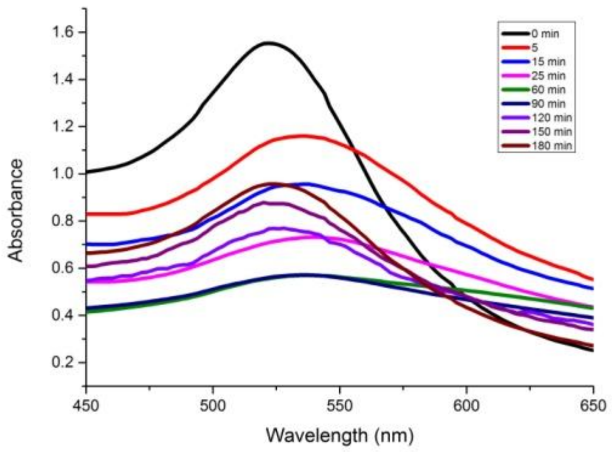

Figure S24. UV-Vis absorption spectra measured every five minutes after the addition of fuel DNA (1.6 μM) in the presence of 6 U/μL enzyme.

3. TEM studies on the structural evolution of core-satellite architectures

7.5 nM DNA4 functionalized 15-AuNPs and 0.5 nM DNA5 functionalized AuNRs were mixed in 100 μL 50 mM Tris-HCl buffer containing 200 mM NaCl, 4 mM $MgCl_2$, and 2 mg/mL BSA. Then 2 U/μL Exo III was added following by the addition of 0.06 nmol fuel DNA 3 into the system to initiate the dissipative assembly of core-satellite architectures between 15-AuNPs and AuNRs. 5 μl of the assembly solution was taken and dropped on TEM grids for each time point, which was dried by filter paper and subjected to TEM imaging.

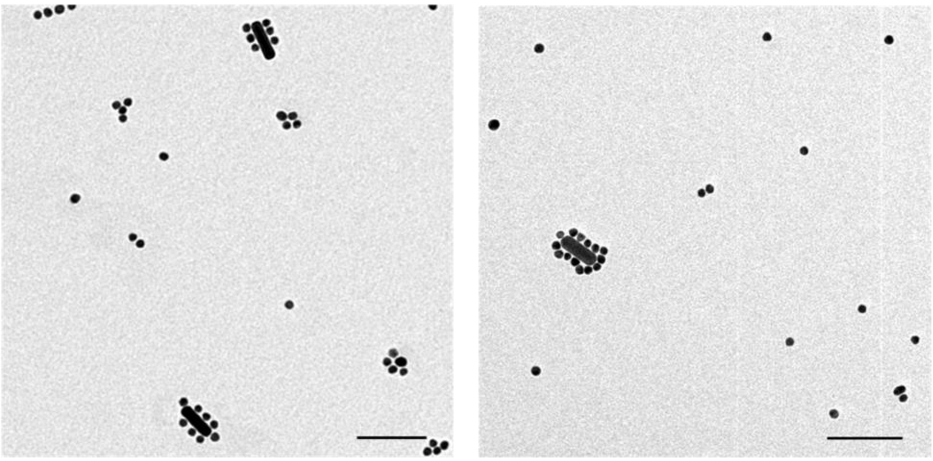

Figure S25. TEM images of core-satellite architectures between AuNRs and 15-AuNPs in the absence of enzyme; scale bars: 100 nm. Samples were taken at 30 min after the addition of fuel DNA.

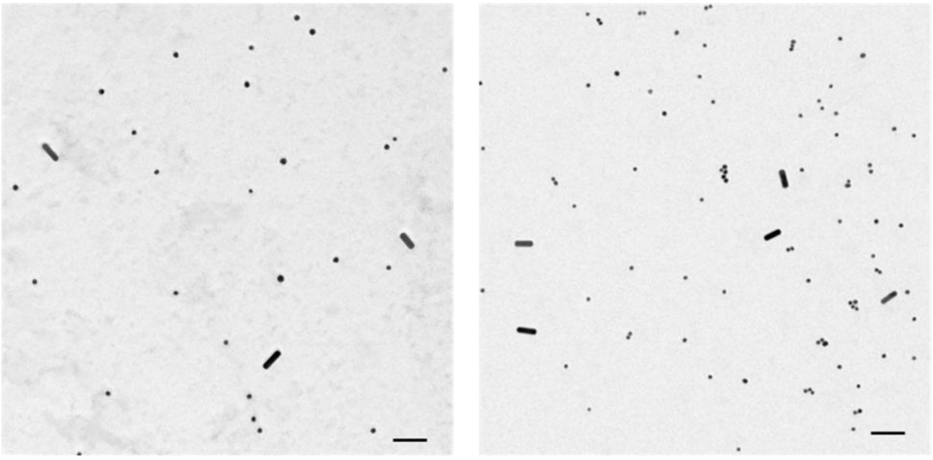

Figure S26. TEM images of core-satellite architectures between AuNRs and 15-AuNPs in the presence of 4 U/μL enzyme; scale bars: 100 nm. Samples were taken at 30 min after the addition of fuel DNA.

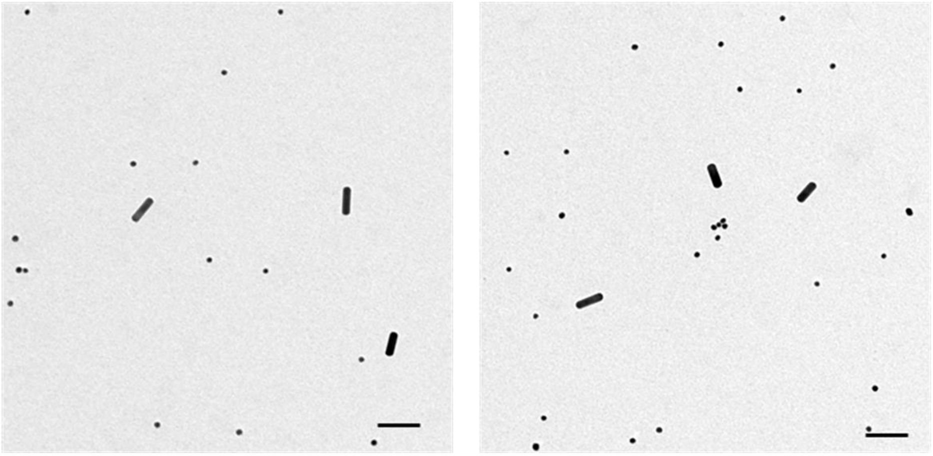

Figure S27. TEM images of core-satellite architectures between AuNRs and 15-AuNPs in the presence of 2 U/μL enzyme; scale bars: 100 nm. Samples were taken immediately after the addition of fuel DNA.

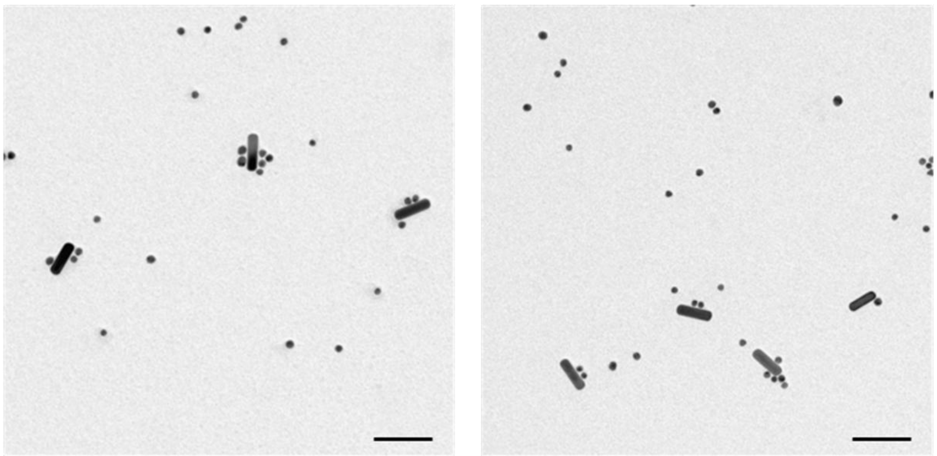

Figure S28. TEM images of core-satellite architectures between AuNRs and 15-AuNPs in the presence of 2 U/μL enzyme; scale bars: 100 nm. Samples were taken at 15 min after the addition of fuel DNA.

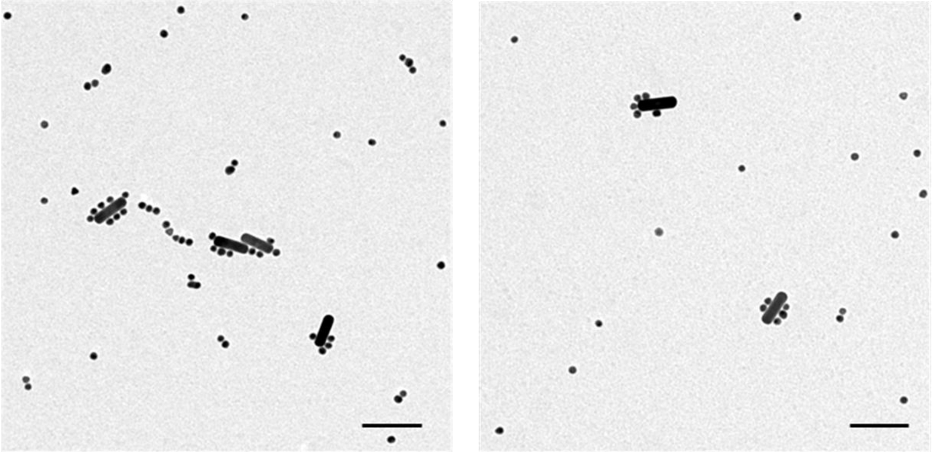

Figure S29. TEM images of core-satellite architectures between AuNRs and 15-AuNPs in the presence of 2 U/μL enzyme; scale bars: 100 nm. Samples were taken at 30 min after the addition of fuel DNA.

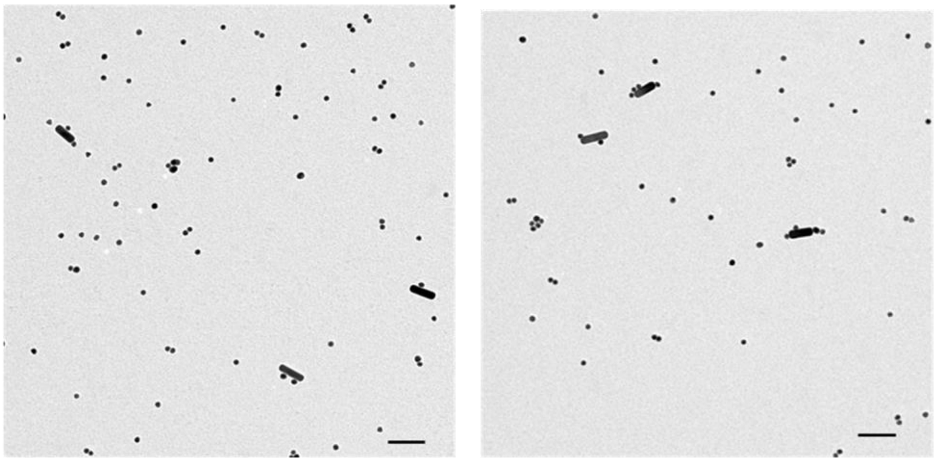

Figure S30. TEM images of core-satellite architectures between AuNRs and 15-AuNPs in the presence of 2 U/μL enzyme; scale bars: 100 nm. Samples were taken at 45 min after the addition of fuel DNA.

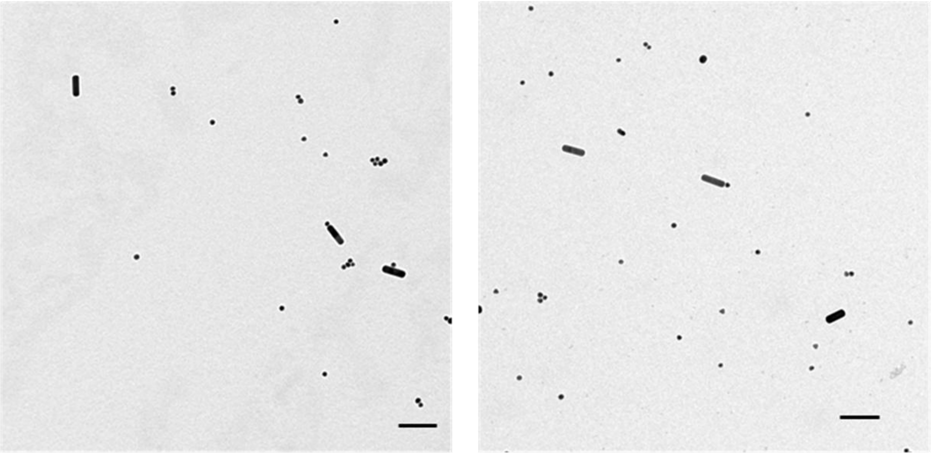

Figure S31. TEM images of core-satellite architectures between AuNRs and 15-AuNPs in the presence of 2 U/μL enzyme; scale bars: 100 nm. Samples were taken at 60 min after the addition of fuel DNA.

TEM studies on the consecutive structural evolution of dissipative core-satellites activated by the sequential addition of two fuel DNA sequences

7.5 nM DNA4 functionalized 15-AuNPs, 0.5 nM DNA5 functionalized AuNRs, and 0.5 nM DNA6 functionalized 55-AuNPs were mixed in 100 μL 50 mM Tris-HCl

buffer containing 200 mM NaCl, 3 mM $MgCl_2$, and 2 mg/mL BSA. Then 3 U/µL EXO III was added. Firstly, 0.06 nmol fuel DNA 3 were added into the system to initiate the dissipative assembly of core-satellite architectures between 15-AuNPs and AuNRs. When the first fuel was consumed at 60 min, the same amount of the second fuel strand DNA 7 (0.06 nmol) was added to induce the dissipative assembly of core-satellite architectures between 15-AuNPs and 55-AuNPs. During this process, 5 µl of the assembly solution was taken and dropped on TEM grids for each time point, which was dried by filter paper and subjected to TEM imaging.

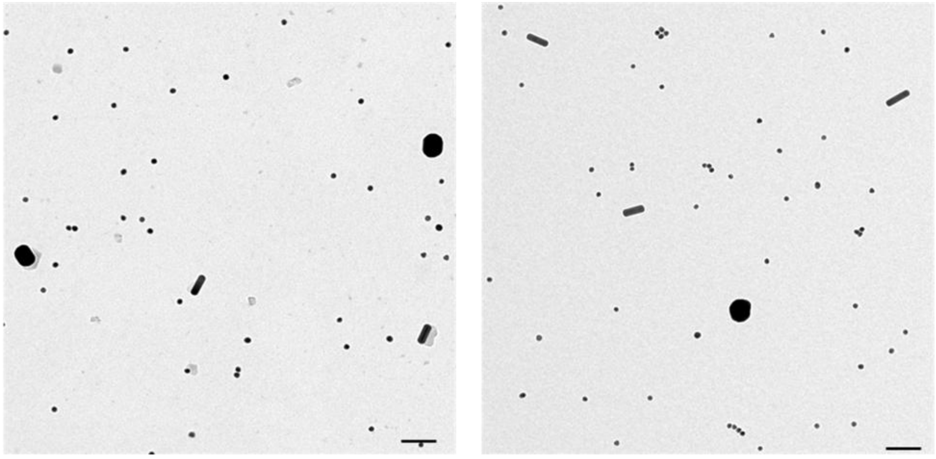

Figure S32. TEM images of core-satellite architectures between 15-AuNPs, AuNRs and 55-AuNPs in the presence of fuel DNA 3 (0 min); scale bars: 100 nm.

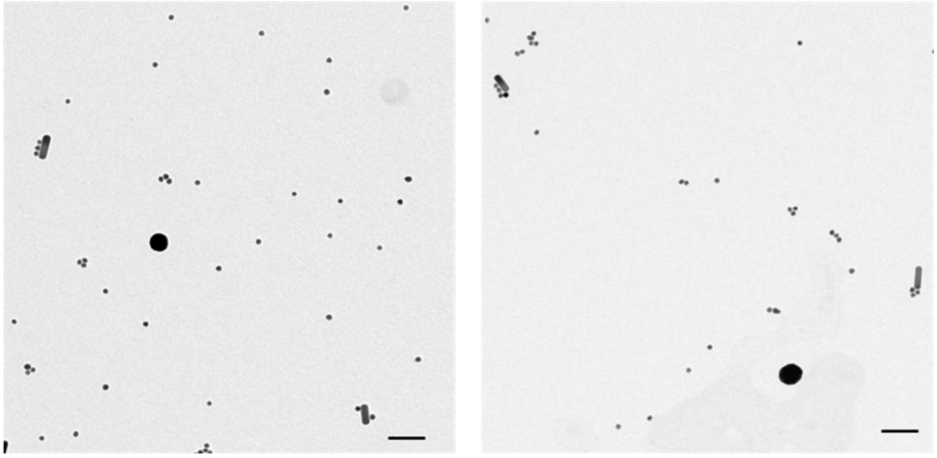

Figure S33. TEM images of core-satellite architectures between 15-AuNPs, AuNRs and 55-AuNPs in the presence of fuel DNA 3 (15 min); scale bars: 100 nm.

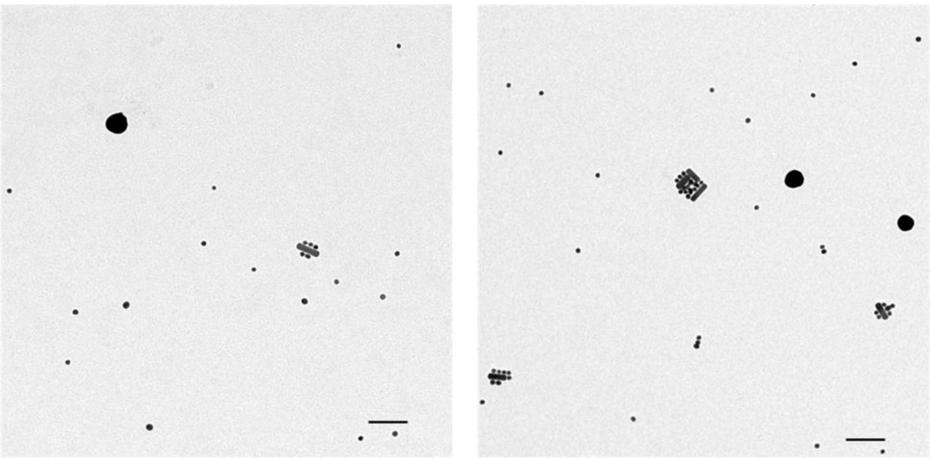

Figure S34. TEM images of core-satellite architectures between 15-AuNPs, AuNRs and 55-AuNPs in the presence of fuel DNA 3 (30 min); scale bars: 100 nm.

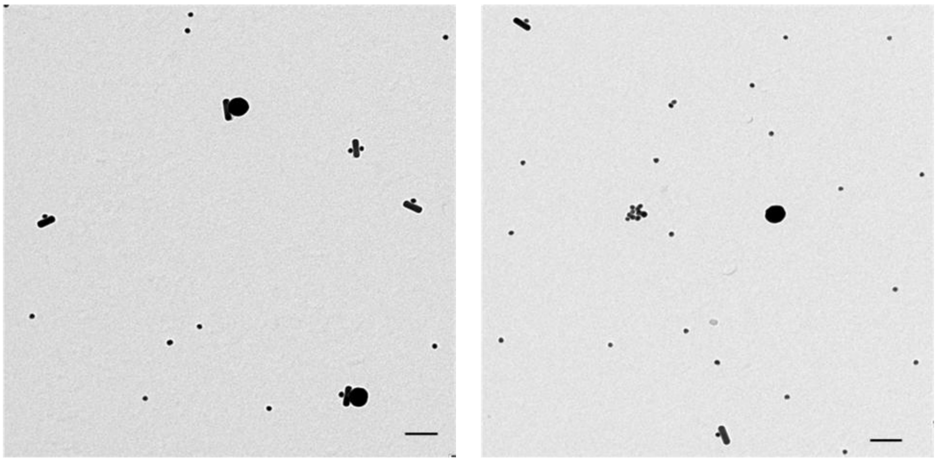

Figure S35. TEM images of core-satellite architectures between 15-AuNPs, AuNRs and 55-AuNPs in the presence of fuel DNA 3 (45 min); scale bars: 100 nm.

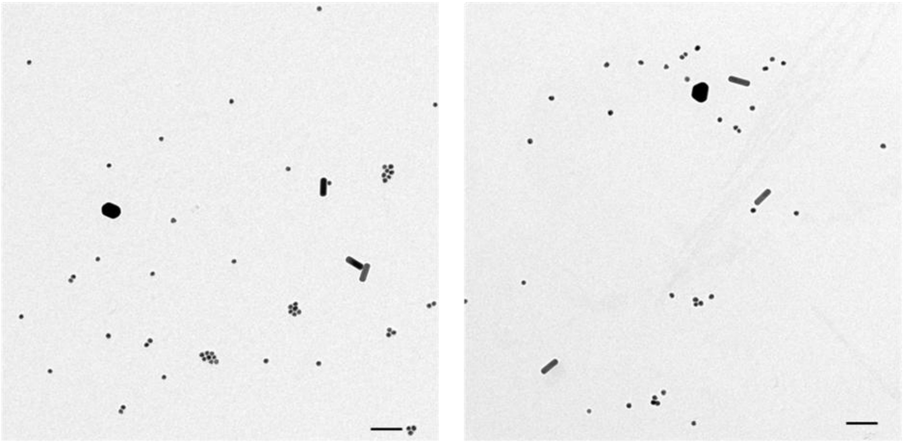

Figure S36. TEM images of core-satellite architectures between 15-AuNPs, AuNRs and 55-AuNPs in the presence of fuel DNA 3 (60 min); scale bars: 100 nm.

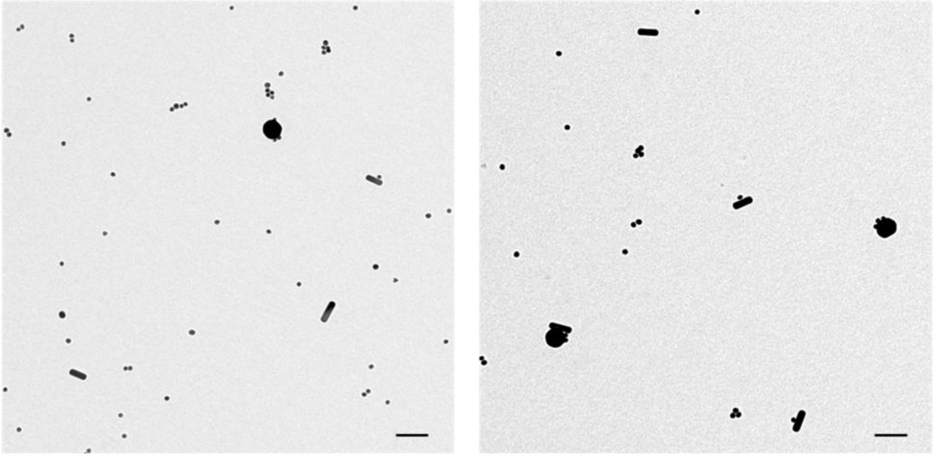

Figure S37. TEM images of core-satellite architectures between 15-AuNPs, AuNRs and 55-AuNPs in the presence of fuel DNA 7 (15 min); scale bars: 100 nm.

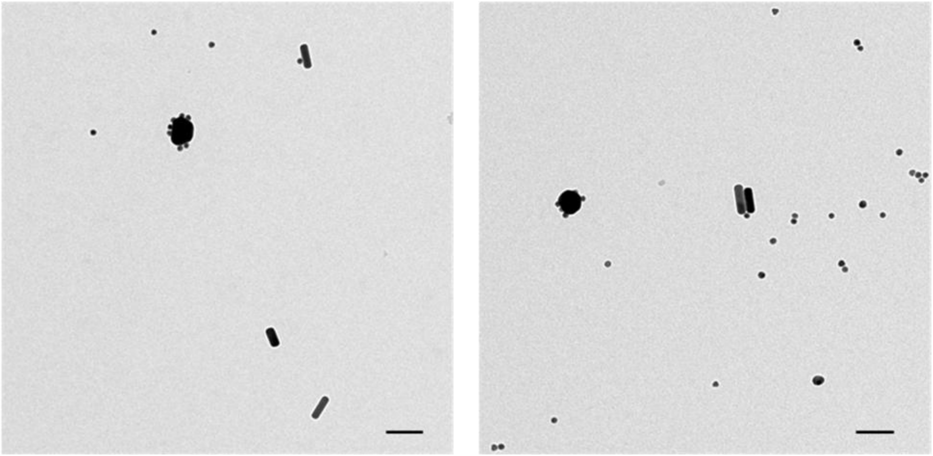

Figure S38. TEM images of core-satellite architectures between 15-AuNPs, AuNRs and 55-AuNPs in the presence of fuel DNA 7 (30 min); scale bars: 100 nm.

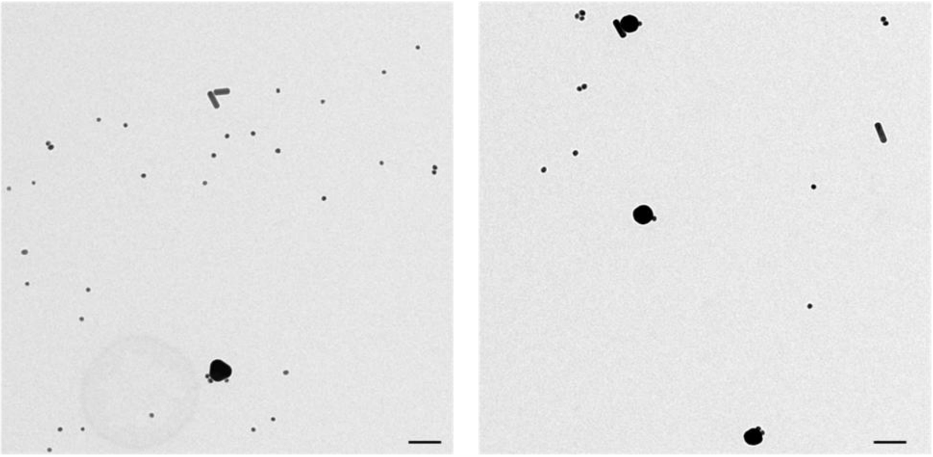

Figure S39. TEM images of core-satellite architectures between 15-AuNPs, AuNRs and 55-AuNPs in the presence of fuel DNA 7 (45 min); scale bars: 100 nm.

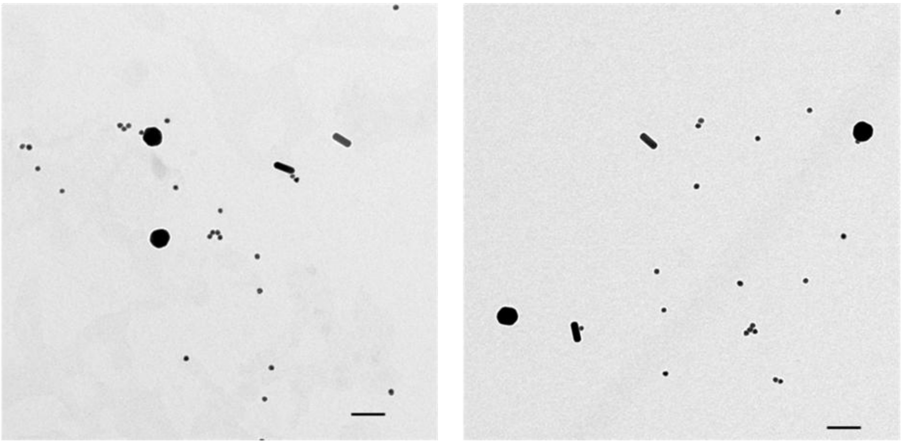

Figure S40. TEM images of core-satellite architectures between 15-AuNPs, AuNRs and 55-AuNPs in the presence of fuel DNA 7 (60 min); scale bars: 100 nm.

4. References

1. Liu, J. & Lu, Y. Preparation of aptamer-linked gold nanoparticle purple aggregates for colorimetric sensing of analytes. *Nat Protoc* **1,** 246–252 (2006).

2. Ziegler, C. & Eychmüller, A. Seeded Growth Synthesis of Uniform Gold Nanoparticles with Diameters of 15−300 nm. *The Journal of Physical Chemistry C* **115**, 4502–4506 (2011)

3. Haiss, W., Thanh, N. T. K., Aveyard, J. & Fernig, D. G. Determination of size and concentration of gold nanoparticles from UV-Vis spectra. *Anal. Chem.* **79**, 4215–4221 (2007).

4. Ohta, S., Glancy, D. & Chan, W. C. DNA-controlled dynamic colloidal nanoparticle systems for mediating cellular interaction. *Science* **351**, 841–845 (2016).

5. Zhang, Z. et al. Mesoporous silica-coated gold nanorods as a light-mediated multifunctional theranostic platform for cancer treatment. *Adv. Mater.* **24**, 1418–1423 (2012).
